# Podocyte injury elicits loss and recovery of cellular forces

**DOI:** 10.1126/sciadv.aap8030

**Published:** 2018-06-27

**Authors:** Kathryn E. Haley, Nils M. Kronenberg, Philipp Liehm, Mustafa Elshani, Cameron Bell, David J. Harrison, Malte C. Gather, Paul A. Reynolds

**Affiliations:** 1 School of Medicine, University of St Andrews, St Andrews KY16 9TF, UK.; 2 Biomedical Sciences Research Complex, University of St Andrews, St Andrews, UK.; 3 Scottish Universities Physics Alliance, School of Physics & Astronomy, University of St Andrews, St Andrews KY16 9SS, UK.

## Abstract

In the healthy kidney, specialized cells called podocytes form a sophisticated blood filtration apparatus that allows excretion of wastes and excess fluid from the blood while preventing loss of proteins such as albumin. To operate effectively, this filter is under substantial hydrostatic mechanical pressure. Given their function, it is expected that the ability to apply mechanical force is crucial to the survival of podocytes. However, to date, podocyte mechanobiology remains poorly understood, largely because of a lack of experimental data on the forces involved. We perform quantitative, continuous, nondisruptive, and high-resolution measurements of the forces exerted by differentiated podocytes in real time using a recently introduced functional imaging modality for continuous force mapping. Using an accepted model for podocyte injury, we find that injured podocytes experience near-complete loss of cellular force transmission but that this loss of force is reversible under certain conditions. The observed changes in force correlate with F-actin rearrangement and reduced expression of podocyte-specific proteins. By introducing robust and high-throughput mechanical phenotyping and by demonstrating the significance of mechanical forces in podocyte injury, this research paves the way to a new level of understanding of the kidney. In addition, in an advance over established force mapping techniques, we integrate cellular force measurements with immunofluorescence and perform continuous long-term force measurements of a cell population. Hence, our approach has general applicability to a wide range of biomedical questions involving mechanical forces.

## Teaser

ERISM continuously maps dynamic changes in the forces podocytes exert on their substrate in a model of acute kidney damage.

## INTRODUCTION

Podocytes serve as the final barrier to protein loss in the kidney during filtration of blood by the glomerulus in vivo. Hence, the podocyte plays an integral role in maintaining the glomerular filtration barrier and preventing protein loss into the urine. Podocyte damage is now recognized as a causal factor in the progression of multiple variants of kidney disease (*1*–*7*). While podocyte adaptation to stress is characterized by cell enlargement and shape remodeling (hypertrophy and foot process effacement) both in vitro (*8*, *9*) and in vivo (*10*–*12*), the mechanisms underlying the pathogenesis of podocyte injury still remain largely unknown. Systemic and intraglomerular hypertension are known effectors of podocyte injury in vivo (*13*–*20*). A direct correlation has been demonstrated between systemic hypertension and concomitant incidence of podocyte depletion (*21*). Increased fluid shear stress and axial capillary wall stress resulting from glomerular hypertension further challenge the podocyte to adapt to increased mechanical stresses, often resulting in podocyte detachment (*22*). Although multiple studies have demonstrated the adaptive stress response of podocytes, wherein cytoskeletal structure is rearranged to increase foot process length while maintaining structural stability of the cell body (*1*, *2*, *8*, *23*), the effects of these structural changes on podocyte mechanobiology remain largely unknown.

The direct correlation between podocyte depletion and the progression of glomerular disease has been elegantly illustrated by Wharram *et al*. (*6*). In a genetically engineered rat model of podocyte depletion, Wharram *et al*. observed transient proteinuria and mesangial sclerosis occurring with 20% podocyte depletion, which progressed to sustained proteinuria and global glomerulosclerosis with podocyte depletion of more than 40% (*6*). Evidence of detached, viable podocytes shed into the urine of patients with diabetic nephropathy (*24*), immunoglobulin A nephropathy (*3*), and focal segmental glomerulosclerosis (*25*, *26*) suggests that impaired podocyte adhesion to the glomerular basement membrane (GBM) may be a pivotal step in the development of glomerulosclerosis. However, to date, there have been limited investigations of the mechanical forces that podocytes apply to their substrate and of the effect of injury on podocyte cellular forces in vitro. This deficiency is, in part, due to a lack of suitable experimental techniques for long-term time-lapse mapping of cellular forces.

Elastic resonator interference stress microscopy (ERISM) is a recently developed functional imaging modality for the mapping of cellular forces; this technique allows continuous monitoring of mechanical cell-substrate interactions for up to several weeks in one experiment, which is impossible or impractical with most established force microscopy methods (*27*). ERISM uses an elastic, optical microcavity as a cell substrate and images cell-induced substrate displacement with nanometer sensitivity by measuring spectral shifts of resonant modes formed inside the microcavity. These displacements are converted into a high-resolution stress map that resolves the focal forces the cell applies to the substrate in different locations. ERISM enables recording of the mechanical activity of cells without acquiring zero-force reference images, eliminating the need to detach cells after a measurement. ERISM therefore drastically simplifies investigation of large numbers of cells on a common substrate and at multiple time points. The microcavity substrates used for ERISM show excellent long-term stability under mechanical stress and standard cell culture conditions, and the optical readout only requires low light intensity, therefore preventing phototoxicity and cell damage. These properties render ERISM particularly well suited for measuring cellular forces over prolonged periods.

Here, we characterized cellular force of both differentiated human and mouse podocytes using ERISM and investigated alterations in podocyte mechanical force during stress response in a puromycin aminonucleoside (PAN) model of glomerular injury in vitro. Our findings demonstrate that dynamic changes in podocyte cellular forces occur in response to acute injury, and further elucidate the role of podocyte force transmission in the progression of glomerular disease.

## RESULTS

### Mapping the force transmission phenotype of differentiated podocytes

**Fig. 1:**
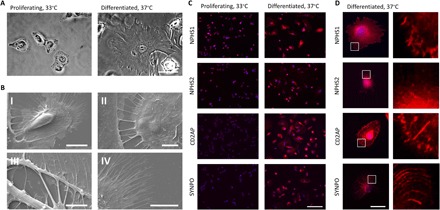
Differentiation of LY podocytes. (**A**) Phase-contrast microscopy of proliferating LY human podocytes cultured at 33°C. Differentiation of LY podocytes at 37°C for 12 days results in a transition to differentiated morphology. Differentiated cells increase in size up to lengths of 150 μm and develop finger-like foot processes around the periphery of the cell. Scale bar, 50 μm. (**B**) Scanning electron microscopy reveals ultrastructures of primary and secondary foot processes on differentiated LY podocytes. (I) LY podocyte differentiated at 37°C for 12 days demonstrates primary foot processes around the cell periphery and extending toward adjacent cell. Scale bar, 20 μm. (II) Primary foot processes interdigitating between differentiated LY podocytes. Scale bar, 5 μm. (III) Primary foot processes branching off of a differentiated LY podocyte. Scale bar, 5 μm. (IV) Secondary foot processes around the outer periphery of a differentiated podocyte. Scale bar, 10 μm. (**C**) Localization and expression of podocyte-specific proteins NPHS1, NPHS2, CD2AP, and SYNPO (red) in proliferating and differentiated LY podocytes assessed by immunocytochemistry at low magnification. DAPI (4′,6-diamidino-2-phenylindole) (blue) detects nuclei. Scale bar, 200 μm. (**D**) Localization and expression of podocyte-specific proteins NPHS1, NPHS2, CD2AP, and SYNPO (red) in a single differentiated LY podocyte. DAPI (blue) detects nuclei. Scale bar, 50 μm; right panels, magnification of area marked by white boxes.

To characterize the force transmission phenotype of differentiated human podocytes, we first assessed the differentiation characteristics of human podocytes in vitro. We used LY human podocytes (a gift from M. Saleem) that contained human telomerase reverse transcriptase (hTERT) and a temperature-sensitive SV40 transgene (*28*). The podocytes were initially cultured in vitro at the permissive temperature of 33°C and then differentiated over the course of 12 days at the nonpermissive temperature of 37°C. Differentiated podocytes demonstrated increased cell size and finger-like foot process extensions, as evidenced by phase-contrast microscopy (Fig. 1A). Ultrastructure of differentiated cells was assessed using scanning electron microscopy, which revealed both primary and secondary foot process extensions branching from the cell body of podocytes (Fig. 1B). We further assessed differentiated cells for expression and localization of the podocyte slit diaphragm proteins nephrin (NPHS1), podocin (NPHS2), and CD2-associated protein (CD2AP) and the foot process protein synaptopodin (SYNPO). Immunofluorescence analysis demonstrated increased distribution of expression of podocyte-specific proteins in differentiated cells compared to proliferating cells (Fig. 1, C and D).

**Fig. 2:**
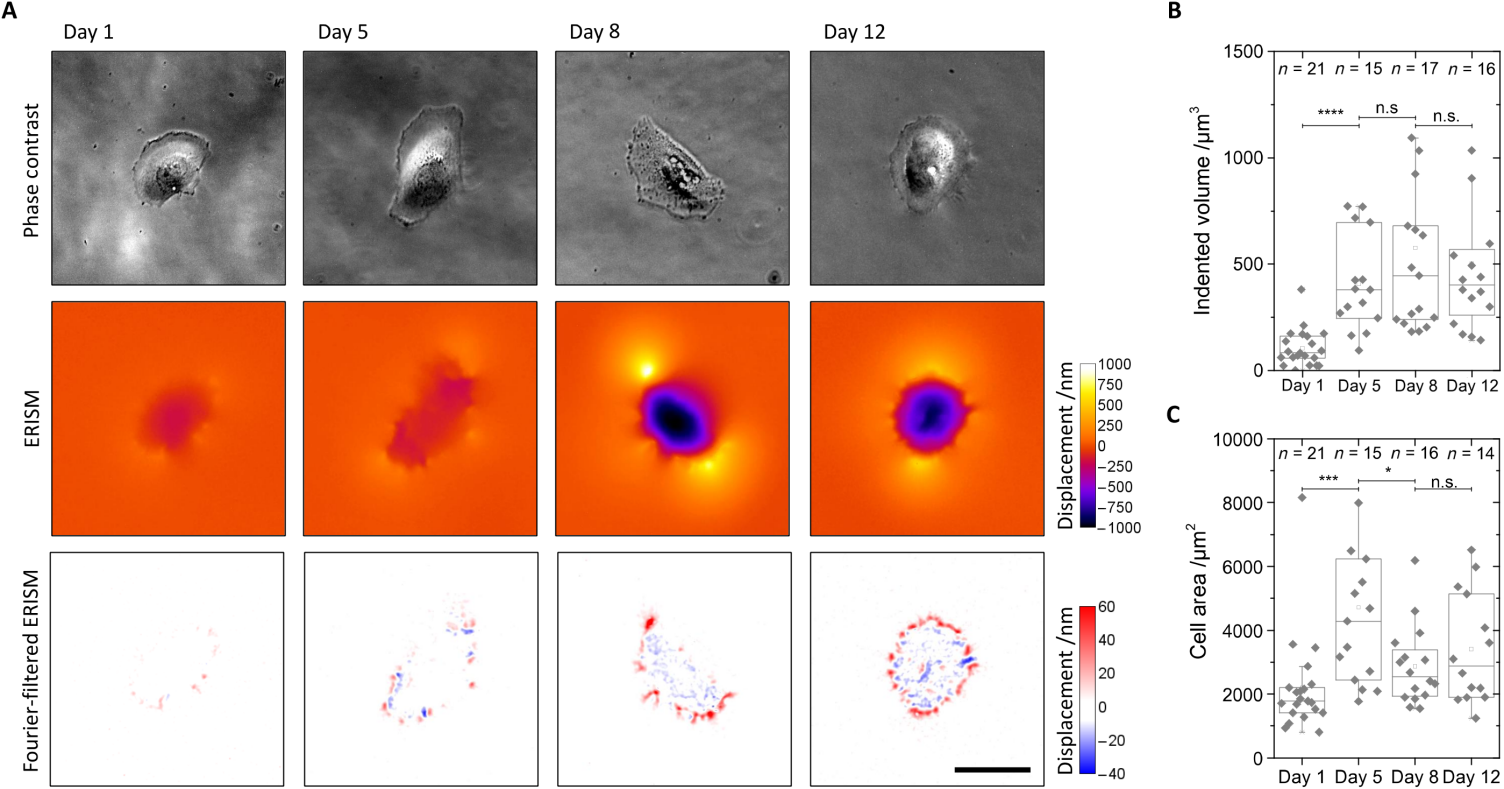
Mapping mechanical force exerted by human podocytes throughout differentiation. (**A**) Representative phase-contrast images (upper row), ERISM displacement maps (middle row), and Fourier-filtered ERISM maps (lower row) taken at different time points during the 12-day differentiation of LY podocytes. (**B** and **C**) Tukey boxplots of (B) mechanical activity and (C) cell area during LY podocyte differentiation. Each dot represents one cell. As data in groups were not normally distributed, groups were compared using the Mann-Whitney *U* test (n.s., no significance; *P < 0.05; ***P < 0.001; ****P < 0.0001). Scale bars, 50 μm.

Having established the human LY podocyte cell line as a model system of healthy differentiated cells in vitro, we then characterized the evolution of cellular forces transmitted by LY podocytes during differentiation on a type IV collagen–coated elastic ERISM substrate with an apparent stiffness of 6.8 kPa (*29*), similar to the stiffness of the GBM. Figure 2A shows a representative example of phase-contrast and ERISM maps recorded over a 12-day differentiation time course. By day 5 of differentiation, LY podocytes demonstrated increased substrate displacement, indicating increased cellular forces. To quantify podocyte cellular forces and confirm statistical significance, we recorded ERISM maps for *n* > 15 cells at days 1, 5, 8, and 12 of differentiation. Taking the total volume by which each cell indents the substrate as a proxy for the overall force applied by the cell, a nearly fivefold increase in median force was observed (indented volume, 82 μm^3^ on day 1 versus 380 μm^3^ on day 5; *P* < 0.0001; Fig. 2B). The increase in force was accompanied by a statistically significant, but less pronounced, increase in median cell area (1800 μm^2^ on day 1 versus 4300 μm^2^ on day 5; *P* < 0.001; Fig. 2C). Between days 6 and 12 of differentiation, the exerted force did not increase further, and the average cell area reduced slightly. However, the pattern of force exertion changed, and an increased number of focal pulling sites were formed at the cell periphery (Fig. 2A). To more clearly visualize force exertion patterns, the broad features from the displacement maps were removed through spatial Fourier filtering of components with spatial frequencies smaller than 0.25 μm^−1^ (Fig. 2A, bottom). This approach revealed focal pulling points (shown in red on the false color map) at the periphery of the cell that were accompanied by focal pushing points (in blue) to their proximal side, that is, a characteristic pull-push twist pattern. Force patterns and the development of forces during differentiation were similar in both clusters of cells and single cells, with both conditions displaying a pull-push pattern. However, in addition, cells seeded on the ERISM substrate at high cell density partly overlap each other, resulting in complex force patterns (fig. S1). Cells that were differentiated on ERISM substrates showed characteristic expression of podocyte-specific proteins (fig. S2).

To determine whether similar force patterns occur in podocytes from other model systems, we analyzed cellular forces during the differentiation of mouse podocytes, using again a temperature-sensitive SV40 transgene to induce differentiation (*30*). By day 12 of differentiation, mouse podocytes demonstrated increased substrate displacement and a nearly fivefold increase in median force (*P* < 0.0001; fig. S3). The successful differentiation of mouse podocytes was again verified by immunofluorescence for podocyte slit diaphragm and foot process proteins (fig. S4).

**Fig. 3:**
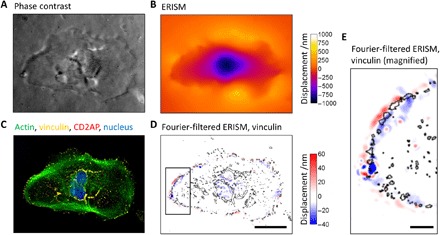
Contractile podocyte forces are colocalized with vinculin expression. (**A**) Phase-contrast image and (**B**) ERISM displacement map generated by a fully differentiated podocyte (day 12). (**C**) Epifluorescence image of the same cell following fixation and immunostaining for actin, vinculin, CD2AP, and nuclear DNA. (**D**) Fourier-filtered ERISM map, with black lines indicating vinculin-rich areas. Scale bar, 50 μm. (**E**) Magnification of the area highlighted in (D). Scale bar, 10 μm.

Immunostaining of differentiated LY podocytes on the ERISM substrate for F-actin, vinculin, and CD2AP revealed a high concentration of vinculin—a protein known to assemble at focal adhesion contacts and in the foot processes of podocytes—at the periphery of the cell and in the center between the pushing and pulling points seen in Fourier-filtered ERISM maps (Fig. 3). Immunostaining for β_1_ integrin revealed colocalization with vinculin and with ERISM maps (fig. S5). These data suggest that the twisting originates from a torque that the contractile forces exerted by the cell induce at the contact points with the substrate as they pull F-actin fibers toward the center of the cell. The number of pull-push twist features increased during the differentiation process, and by day 12, they occurred around the entire periphery of differentiated LY podocytes. Similar to LY podocytes, the number of pull-push twist features also increased in mouse podocytes during the differentiation process, and by day 12, twist features occurred around the periphery of differentiated podocytes (fig. S3). Collectively, these findings demonstrate that ERISM can be used to distinguish and quantify dynamic forces exerted by podocytes during differentiation in vitro.

### Podocyte stress response is reversible in an in vitro PAN injury model

**Fig. 4:**
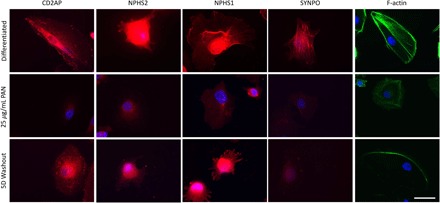
Reduced expression of podocyte-specific proteins in a PAN injury model is reversible in vitro. Localization and expression of podocyte-specific proteins NPHS1, NPHS2, CD2AP, and SYNPO (red) and F-actin (green) in differentiated, PAN (25 μg/ml)–treated LY podocytes and 5-day PAN washout of LY podocytes assessed by immunocytochemistry. DAPI (blue) detects nuclei. Scale bar, 50 μm.

In light of recent findings that detached podocytes in glomerular disease remain viable (*24*, *31*), we hypothesized that acute injury to podocytes by PAN would interfere with the mechanics of cell adhesion but may be reversible. Following 24 hours of PAN treatment, a reduced expression area of podocyte-specific proteins NPHS1, NPHS2, CD2AP, and SYNPO was observed (Fig. 4). Immunofluorescence analysis revealed a loss of focal, punctate staining of both CD2AP and SYNPO, suggesting a loss of slit diaphragm and foot process architecture. Moreover, PAN treatment resulted in rounded cell morphology, with loss of finger-like foot process extensions around the cell periphery, and decreased cell size for some cells. Washout of PAN and return to differentiation medium resulted in a partial recovery of the localization and expression of NPHS1, NPHS2, CD2AP, and SYNPO 5 days after washout. In particular, a return of punctate CD2AP and NPHS1 staining was observed around the cell periphery. Differentiated cell morphology recovered partially, as evidenced by the return of finger-like foot process extensions (Fig. 4). In addition, F-actin immunostaining showed rearrangement and bundling of F-actin in PAN-treated podocytes, which also partially recovered following washout, as evidenced by a return of striated F-actin staining (Fig. 4). Together, these findings suggest that podocyte viability is maintained in some cells following an initial response to acute injury in vitro.

### PAN treatment leads to loss of mechanical force in podocytes

**Fig. 5:**
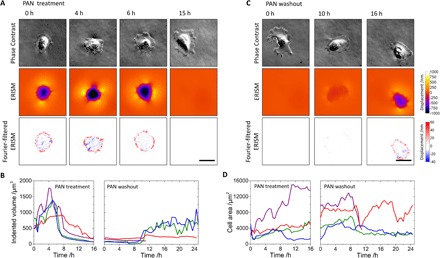
Mapping podocyte force transmission in a PAN injury model. (**A** and **C**) Phase-contrast images (upper rows), ERISM displacement maps (lower rows), and Fourier-filtered ERISM maps (lower rows) during (A) PAN treatment and (C) PAN washout of an LY podocyte. Before the measurement, the cell was allowed to differentiate on the ERISM microcavity substrate over the course of 12 days. (**B** and **D**) Change in exerted cell force (B) and cell area (D) during PAN treatment and PAN washout, continuously measured for four different cells. The green trace corresponds to the cell shown in (A) and (C). The purple trace corresponds to a cell that dies at 11.5 hours, presumably as a result of the PAN injury. Scale bars, 50 µm.

Next, we asked whether PAN injury was concomitant with a loss of podocyte cellular forces. During PAN treatment, podocyte cellular forces were monitored continuously using ERISM. An initial increase in force was observed 4 to 5 hours after the addition of PAN [indented volume, 590 (± 150) μm^3^ at 0 hours versus 1370 (± 350) μm^3^ at 4 hours; *P* = 0.03; Fig. 5, A and B, and video S1]. Decreased podocyte forces were then observed 6.5 hours after the addition of PAN, with a further decrease until 10 to 16 hours after the addition of PAN, when minimal force was detected [indented volume, 130 (± 70) μm^3^] and the push-pull patterns at the periphery of the cells disappeared (forces before PAN treatment versus forces at the end of PAN treatment, *P* = 0.03; peak forces during PAN treatment versus forces at the end of PAN treatment, *P* = 0.03; Fig. 5A). In the absence of PAN treatment, LY podocyte forces oscillated by only 15% (±9% SD; *n* = 7).

Despite the loss in force, phase-contrast microscopy showed continued cell attachment, even after 16 hours of PAN treatment. Consistent with other observations of podocyte adaptation to stress both in vitro and in vivo (*8*, *9*), there was an increase in cell area from an average of 3000 (± 1300) μm^2^ at the onset of PAN treatment to an average of 8000 (± 4900) μm^2^ at 16 hours after PAN addition for three of the four tracked cells (Fig. 5D).

Comparing ERISM and immunofluorescence maps after PAN treatment, we find that podocytes still showed localized vinculin expression, albeit at a reduced level, which explains their ability to adhere to the substrate. However, depending on PAN concentration, the actin cytoskeleton was largely impaired, and only minimal force was detected (fig. S6).

Together, these findings demonstrate that LY podocytes respond to PAN by a near-complete loss of force despite the increase in cell area and the fact that they remain attached to the substrate. This suggests that cell force transmission and cell attachment are separable phenotypes under these conditions.

Similarly, mouse podocyte forces showed an initial increase 1 to 4 hours after the addition of PAN (*P* = 0.04; fig. S7 and video S3). After the 6-hour PAN time point, podocyte forces decreased until 9 to 11 hours after PAN addition, when minimal force was detected and the push-pull patterns at the periphery of the cells disappeared (forces before PAN treatment versus forces at the end of PAN treatment, *P* = 0.004; peak forces during PAN treatment versus forces at the end of PAN treatment, *P* = 0.004; fig. S7). Again, forces oscillated by only 20% before PAN treatment (±10% SD; *n* = 6). Despite the loss of force, phase-contrast microscopy showed continued cell attachment, even after 11 hours of PAN treatment (fig. S7).

### Podocytes recover force transmission following PAN washout

Because podocytes demonstrated partial recovery of localization and expression of NPHS1, NPHS2, CD2AP, and SYNPO after PAN washout, we hypothesized that cellular forces could also be recovered following PAN washout. During PAN washout, partial recovery of podocyte cellular force transmission was observed within the first 24 hours of washout (Fig. 5, B and C, and video S2). The onset of force recovery occurred at 10 hours after PAN washout for three of the four investigated cells, while one cell died during PAN washout. Twenty hours after washout, podocyte forces had recovered to the level of forces exerted by differentiated cells before PAN treatment in two of the four investigated cells, and the pull-push twist patterns at the periphery of the cells returned (Fig. 5C). In contrast, the average cell area did not change significantly in the three cells tracked during PAN washout [4000 (± 2700) μm^2^ at start of washout and 4900 (± 4300) μm^2^ after washout; Fig. 5D]. Forces remained stable and at levels comparable to non–PAN-treated differentiated cells for 3 days after the PAN washout (fig. S8).

A similar partial recovery of cellular forces was observed for mouse podocytes within the first 24 hours following PAN washout (fig. S7 and video S4). The onset of force recovery occurred at 10 hours after PAN washout for four of the six investigated cells, while two cells died during PAN washout. By 24 hours after washout, podocyte forces of the viable cells had recovered to the level of forces exerted by differentiated cells before PAN treatment, and the characteristic pull-push twist patterns at the periphery of the cells returned (fig. S7). Collectively, these findings demonstrate that podocyte force transmission recovers after acute injury within 24 hours of washout in vitro.

## DISCUSSION

Using the ERISM assay on both human and mouse podocytes, we demonstrate continuous monitoring of podocyte cellular forces at high spatial resolution over an extended 17-day time course. ERISM imaging demonstrated contractile forces exerted at localized contact points predominantly around the cell periphery of differentiated podocytes. PAN treatment resulted in membrane blebbing and foot process effacement, as evidenced by F-actin rearrangement and a reduced expression pattern of NPHS1, NPHS2, CD2AP, and SYNPO. PAN treatment also resulted in an initial increase in podocyte cellular forces of up to twofold the original value, followed by a complete loss of cellular force, despite continued cell attachment. Following PAN washout, partial recovery of podocyte force transmission was observed. These findings have important implications for the mechanobiology of podocyte detachment.

The role of mechanical forces in the progression of glomerular disease has previously been explored in the context of systemic and intraglomerular hypertension (*13*–*20*). Intrarenal hypertension and hyperfiltration have long been correlated with glomerular injury (*16*, *17*, *19*). Anderson *et al*. (*13*) were able to show that control of glomerular hypertension limits glomerular injury, findings that were further supported by the hyperfiltration theory published by Brenner *et al*. (*14*), in which control of glomerular pressure was highlighted as a hallmark for the prevention of glomerular disease. However, until recently, the mechanism underlying the role of hypertension in glomerular disease progression remained largely unknown. In a timely review on the role of mechanical forces in the detachment of podocytes, Kriz and Lemley (*22*) hypothesize that detachment of viable podocytes may result from exposure to mechanical distention of the GBM or from fluid shear forces, both resulting in impaired cellular adhesion. In further explaining the role of mechanical forces in podocyte detachment, Endlich *et al*. (*32*) highlight P2X4 and TRPC6 as potential mechanosensor proteins responsible for mechanosignaling in podocytes as well as the mechanosensor talin, which binds both actin and integrins. The authors note that, in single-molecule experiments, talin unfolding results from piconewton forces and this exposes binding sites for vinculin (*32*). Therefore, through talin, vinculin serves as an important actin-integrin connection. It is thus reasonable to suggest that cytoskeletal rearrangement resulting from glomerular hypertension or hypertrophy may initiate a signaling cascade, resulting in loss of α_3_β_1_ integrin cell contacts with the GBM and subsequent podocyte detachment.

Vogelmann *et al*. (*26*) suggest that detached podocytes in glomerular disease are viable and that detachment may be initiated by physiological factors rather than intrinsic podocyte dysregulation. While this hypothesis is supported by numerous studies (*24*, *31*, *33*), it raises the question of the origin of the variability between podocytes that accounts for the detachment of some cells but not others. Hostetter *et al*. (*16*) suggest that podocyte stress response mechanisms may occur through a common pathway, independent of the etiology of the original glomerular injury. On the basis of our findings, we hypothesize that the ability of podocytes to maintain or recover cellular force transmission may be central to preventing detachment.

Despite recent advances in our ability to study podocyte detachment through urinary podocin mRNA/creatinine ratios (*34*, *35*), our current understanding of podocyte detachment in glomerular disease is otherwise limited to histopathological observation (*36*, *37*) and statistical modeling (*38*). Cho *et al*. (*38*) present a quantitative podocyte detachment hypothesis based on the Ising statistical mechanics model that uses Monte Carlo simulations to predict cell attachment under both normal and disease conditions in the glomerulus. We speculate that any condition that results in podocyte injury (that is, changes to the actin cytoskeleton and foot processes) would elicit changes in the pattern of forces applied by podocytes to their substrate. In a clinical setting, we hypothesize that these conditions could include hypertension, diabetic nephropathy, transplant/obesity-related glomerular hypertrophy, or postnephrectomy-related renal changes. In our in vitro experiments using PAN, we find that there is a window of opportunity for recovery within about 24 hours of PAN treatment. Although the physiology in a human kidney is more complex and the extent of clinical damage likely depends on the timing and extent of sustained injury, a similar window of opportunity for recovery may exist in vivo. A clinical example of recovery from podocyte damage is minimal change disease (MCD), in which podocytes remain attached but could exhibit reduced cellular forces. Patients with MCD present with proteinuria but respond well to corticosteroid therapy, in many cases leading to complete remission (*39*).

In conclusion, we investigate here the role of podocyte cellular forces and the underlying mechanobiology preceding podocyte detachment in real time. We show that (i) mechanical forces increase upon differentiation of podocytes, (ii) contractile podocyte forces are transmitted via specific contact points formed in vinculin-rich regions, and (iii) podocyte force transmission is recovered following PAN treatment and washout. On the basis of our investigation of the alterations in mechanical force in a PAN model of glomerular injury, we hypothesize that podocytes are able to recover from acute damage but may remain susceptible to intraglomerular hypertension-induced detachment in vivo. By introducing robust and high-throughput mechanical phenotyping and by demonstrating the significance of mechanical forces in podocyte injury, this research paves the way to a new level of understanding in kidney disease. In addition, our work represents an integration of cellular force measurements with immunofluorescence and of continuous long-term force measurement of a cell population, both of which have been unfeasible or impractical with established force mapping techniques. Hence, our approach has general applicability to a wide range of biomedical questions involving mechanical forces.

## MATERIALS AND METHODS

### Cell culture

LY podocytes were obtained as a gift from M. Saleem (Bristol, UK). LY podocytes were isolated from a 6-year-old female patient with a neuropathic bladder, whose kidney was removed because of infectious complications of the urinary tract but had no evidence of primary glomerular disease. Primary LY podocytes were immortalized via retroviral transduction with the BiCis3 construct, containing hTERT and the SV40 temperature-sensitive transgene (*28*). Proliferating LY podocytes were cultured at the permissive temperature of 33°C, 5% CO_2_ in RPMI supplemented with 10% fetal bovine serum (FBS), 1% penicillin-streptomycin (PS), and 1% insulin-transferrin-selenium (ITS). LY podocytes were cultured under differentiation conditions at 37°C, 5% CO_2_ for 12 days in RPMI supplemented with 2% FBS, 1% PS, and 1% ITS. Mouse podocytes (*30*) were obtained as a gift from N. Jones (Guelph, Ontario, Canada) and were cultured at the permissive temperature of 33°C, 5% CO_2_ in Dulbecco’s modified Eagle’s medium (DMEM)/F12 supplemented with 10% FBS, 1% PS, and recombinant mouse interferon-γ (IFN-γ) (10 U/ml). To induce differentiation, mouse podocytes were cultured under nonpermissive conditions at 37°C, 5% CO_2_ for 12 days in DMEM/F12 supplemented with 2% FBS and 1% PS, in the absence of IFN-γ. PAN was used as a glomerular injury model at a concentration of 25 μg/ml for 24 hours. PAN washout experiments entailed 2× phosphate-buffered saline (PBS) wash and replacement of PAN with fresh differentiation medium for 5 days. Medium was changed every 2 to 3 days for all experiments.

### Scanning electron microscopy

LY podocytes were cultured on No.2 glass coverslips for 12 days at 37°C, 5% CO_2_ in RPMI supplemented with 2% FBS, 1% PS, and 1% ITS. Fully differentiated cells were washed in PBS and fixed overnight (>12 hours) at 4°C with 2.5% glutaraldehyde in 0.1 M phosphate buffer (pH 7.2). Cells were washed three times for 5 min in 0.1 M phosphate buffer (pH 7.2), shaking at 15 rpm. Cells were dehydrated in 70, 80, and 90% ethanol for 10 min, respectively, shaking at 15 rpm. Cells were washed three times for 10 min in 100% ethanol followed by a 10-min wash in hexamethyldisilazane and dried overnight. Coverslips were then sputter-coated in gold with a Q150T Turbo-Pumped Sputter Coater and imaged with an EVO MA10 scanning electron microscope.

### Immunocytochemistry

Podocytes were cultured on No.2 glass coverslips in six-well plates or directly on ERISM chips, where immunofluorescence was combined with force measurements. Cells were washed two times in PBS and fixed in 4% paraformaldehyde for 10 min at room temperature. Cells were washed for 5 min in PBS and permeabilized in PBS supplemented with 0.2% Triton X-100 for 5 min, shaking at 15 rpm. Cells were then blocked with 5% bovine serum albumin (BSA) and 0.1% Triton X-100 in PBS for 30 min, shaking at 15 rpm. Coverslips were incubated overnight at 4°C in primary antibody: nephrin rabbit polyclonal antibody (1:200) (Thermo Fisher Scientific, PA5-20330), CD2AP rabbit polyclonal antibody (1:200) (Sigma-Aldrich, HPA003326), podocin mouse polyclonal antibody (1:150) (Abcam, ab168625), synaptopodin mouse monoclonal antibody (1:200) (Progen Biotechnik, 61094), β_1_ integrin rabbit polyclonal antibody (1:400) (Proteintech, 12594-1-AP), or vinculin mouse monoclonal antibody (1:200) (Sigma-Aldrich, FAK100) in 5% BSA, 0.1% Triton X-100 in PBS. Following incubation in primary antibody, coverslips were washed three times for 5 min in PBS, shaking at 15 rpm. Coverslips were incubated in secondary antibody (1:200) Alexa Fluor 568 goat anti-mouse (Life Technologies, Z25006), Alexa Fluor 568 goat anti-rabbit (Life Technologies, Z25306), or Alexa Fluor 647 phalloidin (Thermo Fisher Scientific, A22287), where indicated, in 3% BSA in PBS for 60 min protected from light. Coverslips were then washed five times for 5 min in PBS protected from light, shaking at 15 rpm. Coverslips were mounted on frosted glass slides with ProLong Gold Antifade Reagent with DAPI (Invitrogen, P36931). Slides were imaged with a Leica DM5500 B microscope.

### Elastic resonator interference stress microscopy

ERISM substrates were fabricated, as described previously (*27*), and a silicon chamber (surface area, 1.60 × 1.60 cm^2^; Ibid) was applied. Substrates were coated with type IV collagen at 4°C overnight (Sigma-Aldrich, CC5533), washed two times with PBS, and preincubated at 33°C, 5% CO_2_ with fresh RPMI medium supplemented with 10% FBS, 1% PS, and 1% ITS for 30 min. LY podocytes were then seeded onto the ERISM substrate at a density of 500 cells per well. Cells were cultured at the permissive temperature of 33°C, 5% CO_2_ for 24 hours and then transferred to 37°C, 5% CO_2_ culture conditions in RPMI supplemented with 2% FBS, 1% PS, and 1% ITS for 12 days during differentiation. Mouse podocytes were seeded on ERISM substrates and maintained at the nonpermissive conditions as described above. To obtain single-cell measurement for the later stages of differentiation, mouse podocytes were differentiated for 8 days before seeding on ERISM substrates. ERISM force measurements were performed as described in (*27*). ERISM maps were recorded on days 1, 5, 8, and 12 of differentiation (using a 20× microscope objective) (*n* ≥ 15) and continuously (one frame every 30 min for each of the fields of view that were tracked) for the duration of PAN treatment and the first 24 hours of PAN washout (*n* = 4). To record time-lapse data for multiple cells simultaneously, the previously described ERISM configuration was extended by a programmable motorized translation stage that facilitated monitoring multiple regions of interest in quick succession.

### Statistical analysis

The Kolmogorov-Smirnov normality test was used to investigate whether data in groups followed Gaussian distribution. As data in groups were not normally distributed, groups were compared using the Mann-Whitney *U* test (*P < 0.05; **P < 0.01; ***P < 0.001; ****P < 0.0001).

## Supplementary Material

http://advances.sciencemag.org/cgi/content/full/4/6/eaap8030/DC1

## SUPPLEMENTARY MATERIALS

Supplementary material for this article is available at http://advances.sciencemag.org/cgi/content/full/4/6/eaap8030/DC1 fig. S1. Mapping mechanical force exerted by an LY podocyte layer throughout differentiation. fig. S2. Differentiation of LY podocytes on ERISM substrate. fig. S3. Mapping mechanical force exerted by mouse podocytes throughout differentiation. fig. S4. Differentiation of mouse podocytes. fig. S5. Contractile podocyte forces are colocalized with vinculin expression. fig. S6. Mapping force transmission and vinculin expression in a PAN injury model. fig. S7. Mapping mouse podocyte force transmission in a PAN injury model. fig. S8. Comparison of LY podocyte force before and after PAN treatment and washout. video S1. Addition of PAN to differentiated LY podocytes. video S2. Washout of PAN-treated, differentiated LY podocytes. video S3. Addition of PAN to differentiated mouse podocytes. video S4. Washout of PAN-treated, differentiated mouse podocytes.
