# Supplementary material for: Podocyte injury elicits loss and recovery of cellular forces
Source: Sci Adv. 2018 Jun 27;4(6):eaap8030. doi: 10.1126/sciadv.aap8030 (PMC6021140; doi:10.1126/sciadv.aap8030)
Supplement: http://advances.sciencemag.org/cgi/content/full/4/6/eaap8030/DC1 [file supp_4_6_eaap8030__index.html]

Science Advances | Science Advances

## Supplementary Materials

**This PDF file includes:**

- fig. S1. Mapping mechanical force exerted by an LY podocyte layer throughout differentiation.
- fig. S2. Differentiation of LY podocytes on ERISM substrate.
- fig. S3. Mapping mechanical force exerted by mouse podocytes throughout differentiation.
- fig. S4. Differentiation of mouse podocytes.
- fig. S5. Contractile podocyte forces are colocalized with vinculin expression.
- fig. S6. Mapping force transmission and vinculin expression in a PAN injury model.
- fig. S7. Mapping mouse podocyte force transmission in a PAN injury model.
- fig. S8. Comparison of LY podocyte force before and after PAN treatment and washout.

Download PDF

**Other Supplementary Material for this manuscript includes the following:**

- video S1 (.avi format). Addition of PAN to differentiated LY podocytes.
- video S2 (.avi format). Washout of PAN-treated, differentiated LY podocytes.
- video S3 (.avi format). Addition of PAN to differentiated mouse podocytes.
- video S4 (.avi format). Washout of PAN-treated, differentiated mouse podocytes.

**Files in this Data Supplement:**

- Adobe PDF - aap8030\_SM.pdf
