# Supplementary material for: Podocyte injury elicits loss and recovery of cellular forces
Source: Sci Adv. 2018 Jun 27;4(6):eaap8030. doi: 10.1126/sciadv.aap8030 (PMC6021140; doi:10.1126/sciadv.aap8030)
Supplement: http://advances.sciencemag.org/cgi/content/full/4/6/eaap8030/DC1 [file aap8030_SM.pdf]

Science Advances

AAAS

advances.sciencemag.org/cgi/content/full/4/6/eaap8030/DC1

Supplementary Materials for

Podocyte injury elicits loss and recovery of cellular forces

Kathryn E. Haley, Nils M. Kronenberg, Philipp Liehm, Mustafa Elshani, Cameron Bell, David J. Harrison, Malte C. Gather, Paul A. Reynolds

Published 27 June 2018, *Sci. Adv.* **4**, eaap8030 (2018)
DOI: 10.1126/sciadv.aap8030

The PDF file includes:

- fig. S1. Mapping mechanical force exerted by an LY podocyte layer throughout differentiation.
- fig. S2. Differentiation of LY podocytes on ERISM substrate.
- fig. S3. Mapping mechanical force exerted by mouse podocytes throughout differentiation.
- fig. S4. Differentiation of mouse podocytes.
- fig. S5. Contractile podocyte forces are colocalized with vinculin expression.
- fig. S6. Mapping force transmission and vinculin expression in a PAN injury model.
- fig. S7. Mapping mouse podocyte force transmission in a PAN injury model.
- fig. S8. Comparison of LY podocyte force before and after PAN treatment and washout.

Other Supplementary Material for this manuscript includes the following:
(available at advances.sciencemag.org/cgi/content/full/4/6/eaap8030/DC1)

- video S1 (.avi format). Addition of PAN to differentiated LY podocytes.
- video S2 (.avi format). Washout of PAN-treated, differentiated LY podocytes.
- video S3 (.avi format). Addition of PAN to differentiated mouse podocytes.
- video S4 (.avi format). Washout of PAN-treated, differentiated mouse podocytes.

Supplementary Figures

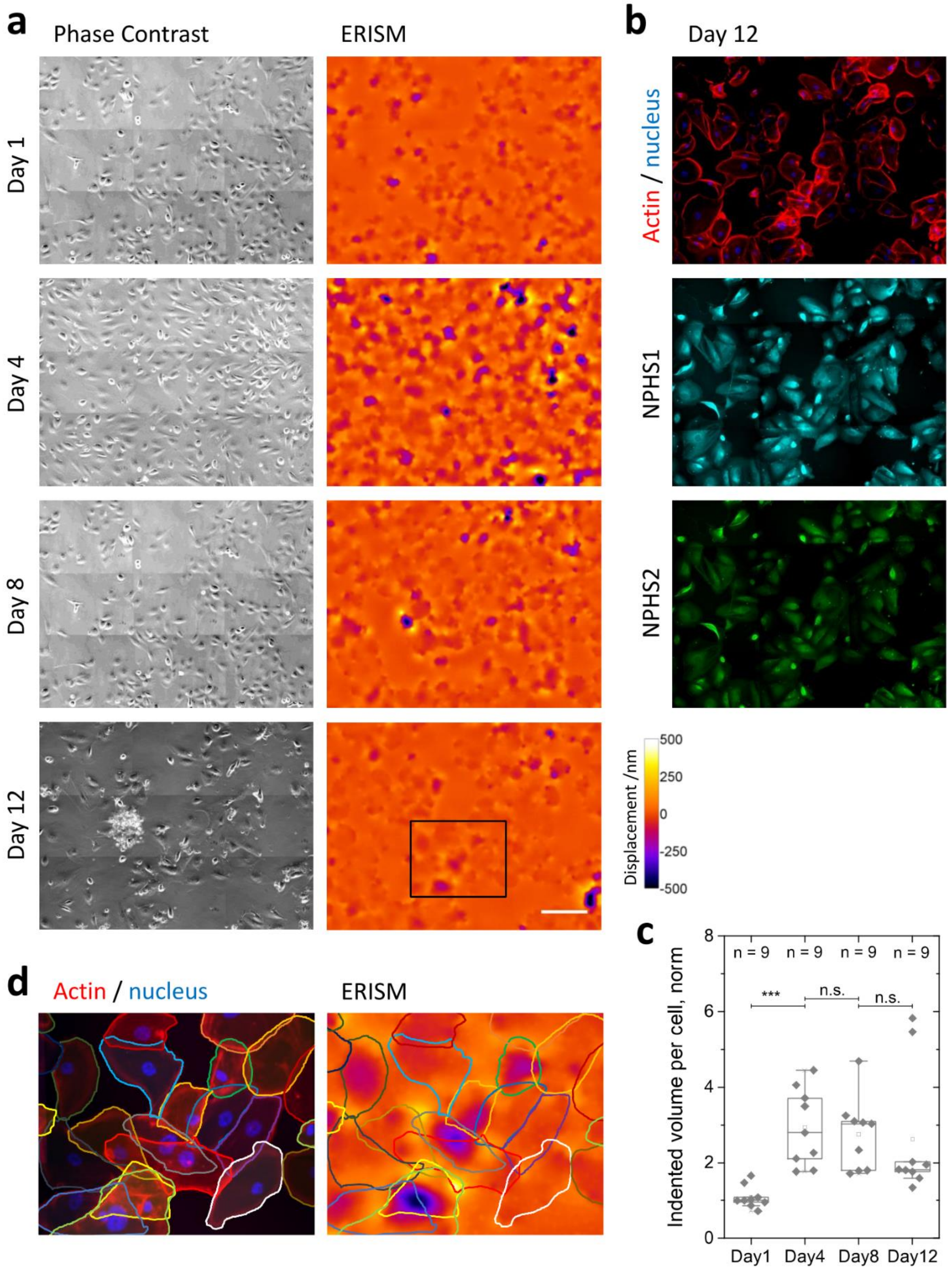

fig. S1. Mapping mechanical force exerted by an LY podocyte layer throughout differentiation. (a) Phase contrast images (left) and ERISM displacement maps (right) taken at different time-points during the 12-day differentiation of LY podocytes that are in contact with each other. (b) Localization and expression of F-actin (top) and podocyte-specific proteins NPHS1 (middle) and NPHS2 (bottom) in differentiated LY podocytes showing the same field of view as in (a) at Day 12. DAPI (blue) detects nuclei. (c) Mechanical activity

during LY podocyte differentiation. Tukey boxplot of the mean indented volume per cell for the ERISM displacement maps shown in (a) normalized to the median indented volume on Day 1. Each dot represents one of the nine fields of view stitched together in (a). As data in groups was not normally distributed, groups were compared using the Mann-Whitney U-test (n.s.: no significance; ***$p < 0.001$). (d) Blow-up of F-actin and nuclei expression (left) and ERISM displacement map (right) of the area highlighted in (a). Fully differentiated podocytes partly overlap with each other and show a complex force pattern. The contours of individual cells are marked in different colors. Scale bar, 200 μm.

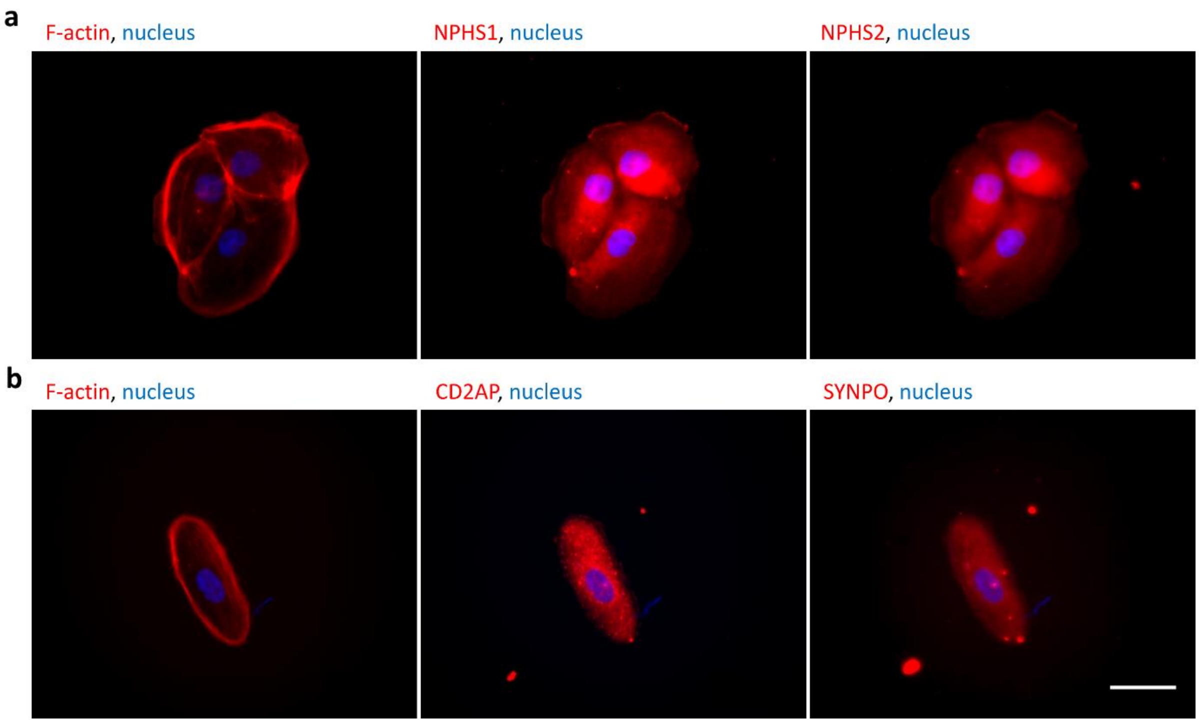

fig. S2. Differentiation of LY podocytes on ERISM substrate. (**a**) Localization and expression of F-actin and podocyte-specific proteins NPHS1 and NPHS2. (**b**) Localization and expression of F-actin and podocyte-specific proteins CD2AP and SYNPO. DAPI (blue) detects nuclei. LY Podocytes were differentiated at 37°C on soft ERISM chips for 12 days. Scale bar, 50 µm.

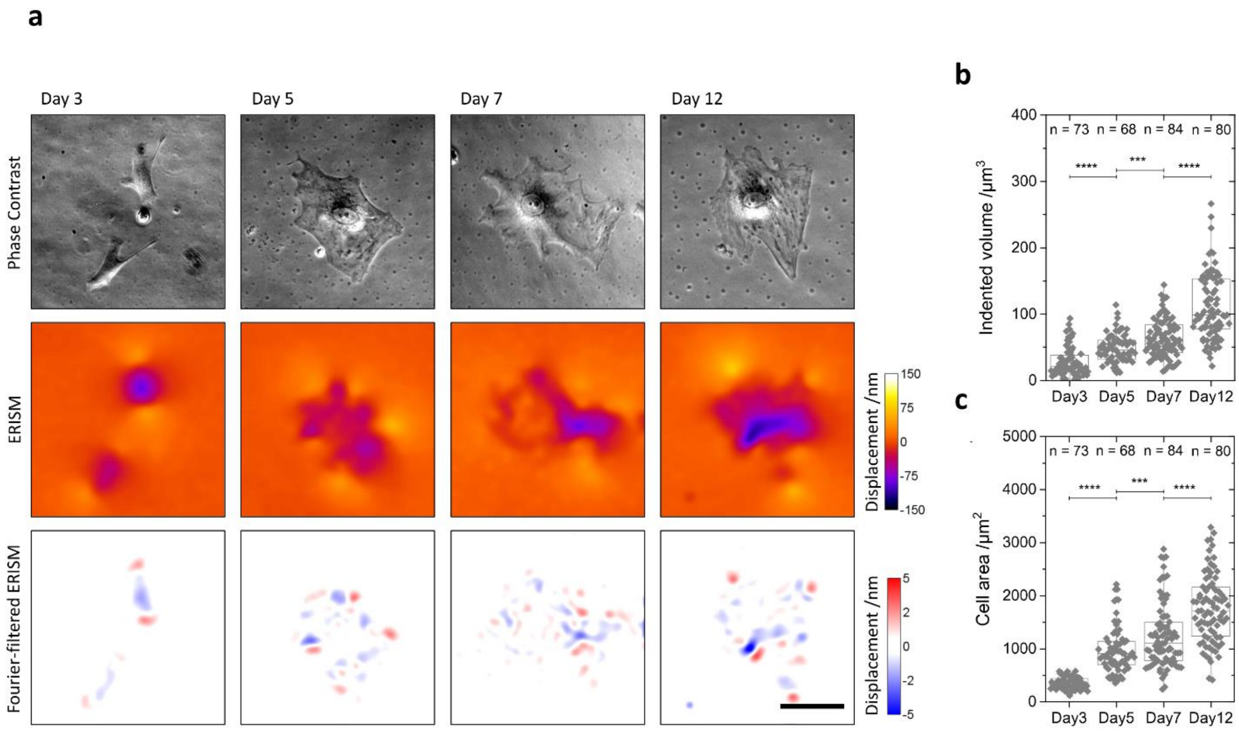

fig. S3. Mapping mechanical force exerted by mouse podocytes throughout differentiation. (a) Representative phase contrast images (upper row), ERISM displacement maps (middle row), and Fourier-filtered ERISM maps (lower row) taken at different time-points during the 12-day differentiation of mouse podocytes. (b,c) Tukey boxplots of (b) mechanical activity and (c) cell area during mouse podocyte differentiation. Each dot represents one cell. As data in groups was not normally distributed, groups were compared using the Mann-Whitney U-test (n.s.: no significance, ***p < 0.001; ****p < 0.0001). Scale bars, 50 µm.

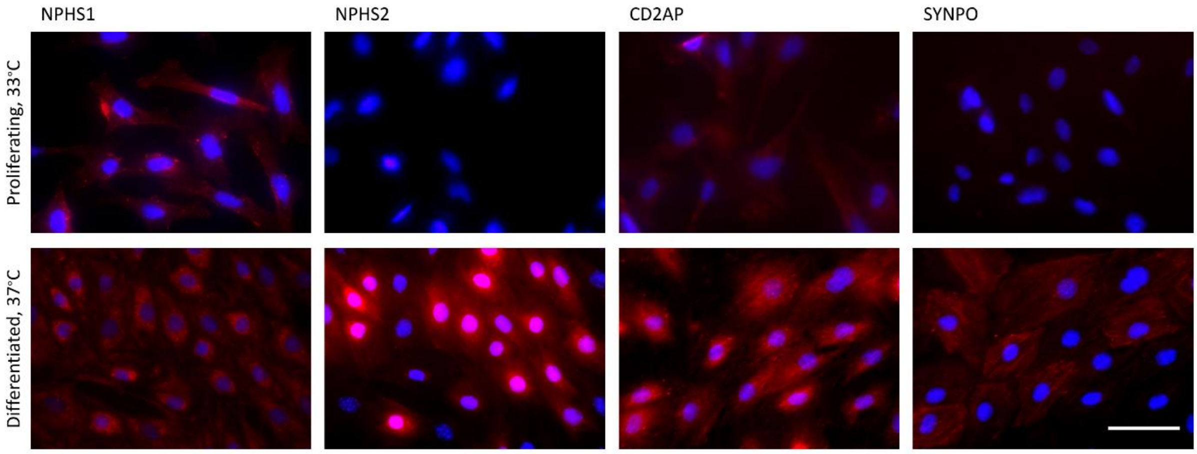

fig. S4. Differentiation of mouse podocytes. Localization and expression of podocyte-specific proteins NPHS1, NPHS2, CD2AP and SYNPO (red) in proliferating and differentiated mouse podocytes assessed by immunocytochemistry. DAPI (blue) detects nuclei. Scale bar, 200 μm.

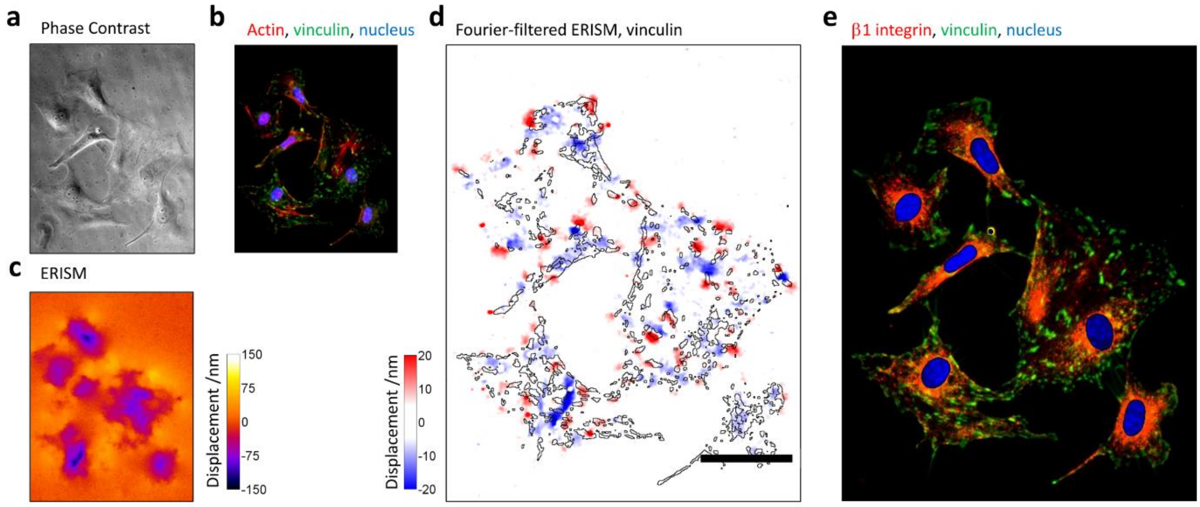

fig. S5. Contractile podocyte forces are colocalized with vinculin expression. (a) Phase contrast image, (b) epi-fluorescence image of immunostaining for actin, vinculin, and nuclear DNA and (c) ERISM displacement map generated by fully differentiated mouse podocytes (Day12). (d) Fourier-filtered ERISM map of same field of view as in (a)-(c), with black lines indicating vinculin-rich areas. (e) Epi-fluorescence image of immunostaining for vinculin, and β1 integrin and nuclear DNA reveals co-localization of vinculin and β1 integrin. Scale bar, 50 μm.

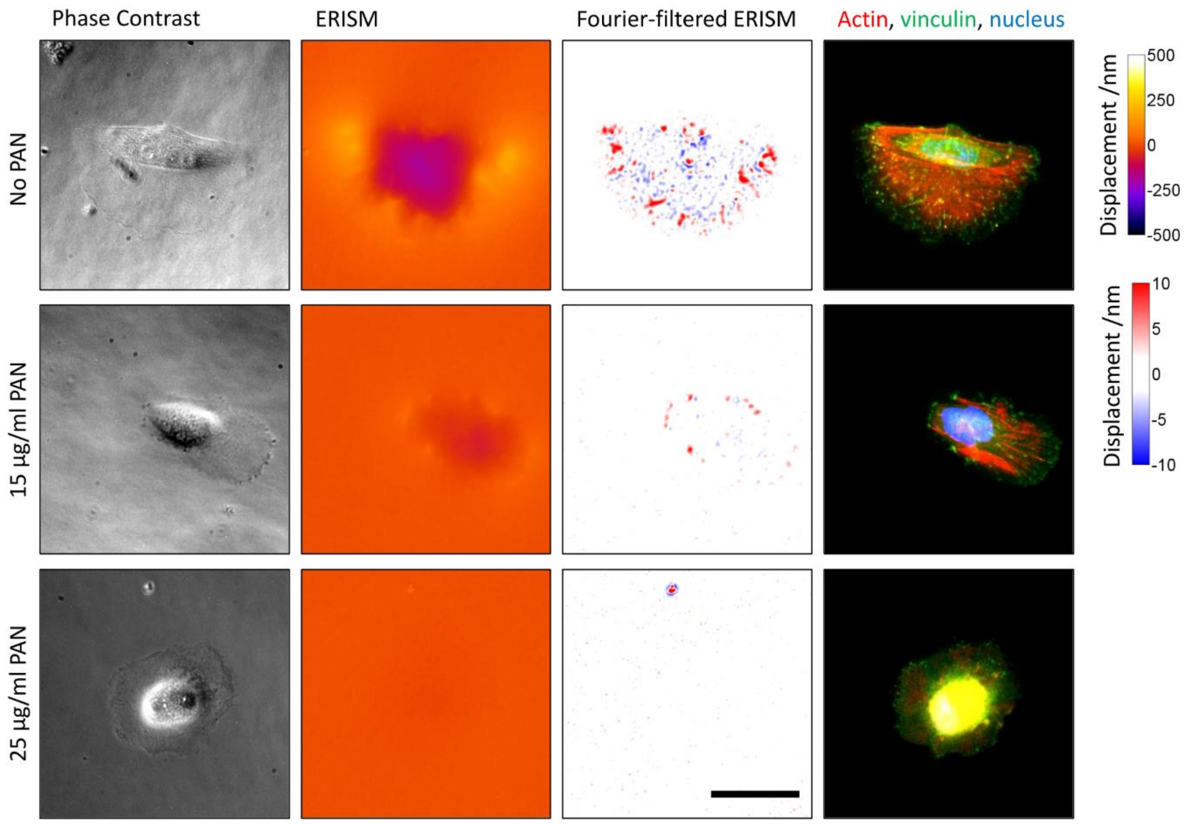

fig. S6. Mapping force transmission and vinculin expression in a PAN injury model. Phase contrast image, ERISM displacement map generated by a fully differentiated podocyte, Fourier-filtered ERISM map and epi-fluorescence image of same cells following fixation and immunostaining for actin, vinculin, and nuclear DNA. Cells were not treated (upper panels) or treated with either 15 µg/ml PAN (middle panels) or 25 µg/ml PAN (lower panels) for 9 hours. Scale bar, 50 µm.

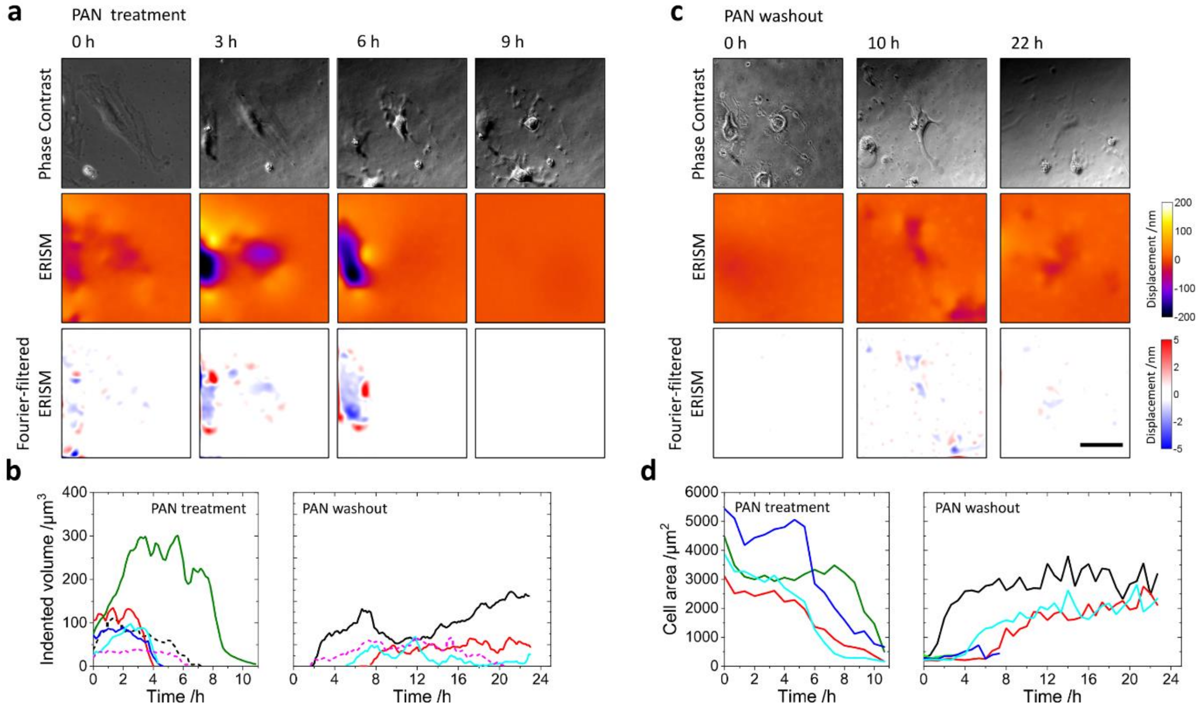

fig. S7. Mapping mouse podocyte force transmission in a PAN injury model. (a,c) Phase contrast images (upper rows), ERISM displacement maps (lower rows), and Fourier-filtered ERISM maps (lower rows) during (a) PAN treatment and (c) PAN washout of a mouse podocyte. Prior to the measurement, the cell was allowed to differentiate on the ERISM microcavity substrate over the course of 12 days. (b,d) Change in exerted cell force (b) and cell area (d) during PAN treatment and PAN washout, continuously measured for six different cells. Dashed lines represent cells that were not fully in the field of view. The cyan trace corresponds to the cell shown in (a) and (c). Scale bar, 50 μm.

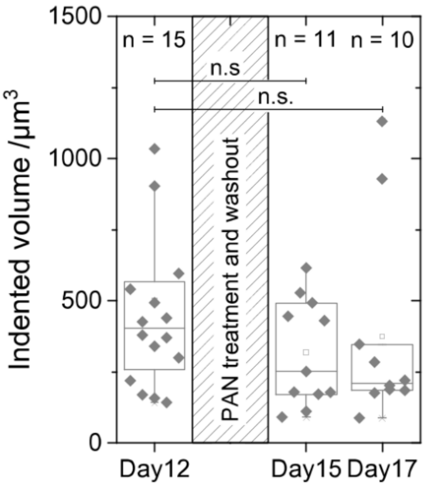

fig. S8. Comparison of LY podocyte force before and after PAN treatment and washout. After the PAN treatment and washout experiment presented in Fig. 5 of the main text, the exerted cell force was followed for two more days (Day 15 and 17). The forces were maintained at the level of pre-PAN treatment (Day 12) during this time. Each dot represents one cell. Data shown in Tukey boxplot. As data in groups was not normally distributed, groups were compared using the Mann-Whitney U-test (n.s.: no significance).
